# On the Number of Close-to-Optimal Feature Sets

**Published:** 2007-02-16

**Authors:** Edward R. Dougherty, Marcel Brun

**Affiliations:** 1 Department of Electrical and Computer Engineering, Texas A & M University, College Station, TX.; 2 Computational Biology Division, Translational Genomics Research Institute, Phoenix, AZ.

**Keywords:** classification, covariance model, feature sets, gene expression

## Abstract

The issue of wide feature-set variability has recently been raised in the context of expression-based classification using microarray data. This paper addresses this concern by demonstrating the natural manner in which many feature sets of a certain size chosen from a large collection of potential features can be so close to being optimal that they are statistically indistinguishable. Feature-set optimality is inherently related to sample size because it only arises on account of the tendency for diminished classifier accuracy as the number of features grows too large for satisfactory design from the sample data. The paper considers optimal feature sets in the framework of a model in which the features are grouped in such a way that intra-group correlation is substantial whereas inter-group correlation is minimal, the intent being to model the situation in which there are groups of highly correlated co-regulated genes and there is little correlation between the co-regulated groups. This is accomplished by using a block model for the covariance matrix that reflects these conditions. Focusing on linear discriminant analysis, we demonstrate how these assumptions can lead to very large numbers of close-to-optimal feature sets.

## Introduction

Concern has recently been expressed regarding the fact that different studies reveal different gene sets for microarray-based cancer classifiers meant to achieve a decision for the same characteristics. In particular, we refer to the concern regarding different gene sets for predicting prognosis for breast cancer (Ioannidis, 2005; Jenssen and Hovig, 2005). In one study, the issue is addressed by repeatedly randomly selecting a subset of the data, designing a classifier on the subset, estimating the error of the designed classifier on the remaining data points, and considering the distribution of the genes composing the various classifier feature sets (Michiels et al. 2005). Concern is expressed over the wide variability of the feature sets and the large number of genes contributing to the various feature sets. Reference is made to the sample size. While sample size is a factor, it is not the only one. A wide array of feature sets can occur with large samples owing to the relations among the genes. The potential for a large number of “best” feature sets is an inherent property of pattern recognition and is not particular to microarrays. In this paper we will explore this potential taking a model-based approach, specifically, modeling the covariance matrix of the features.

Before proceeding we must clarify the problem. Suppose we have some large number, *D*, of features from which to choose, say 20,000 gene-expression levels from a microarray. If we know the class conditional probability distributions, say the distributions of the genes for two phenotypes, then the collection of all *D* features is optimal. Many may be redundant and can be removed with negligible effect on classification accuracy; nonetheless, all can be kept without doing harm to the classification accuracy. This, however, is abstract. In practice, feature sets and classifiers must be derived from sample data, and here there is the common situation of the error declining as the number of features grows to a certain point and then beginning to increase as the number of features increases beyond that point – the *peaking phenomenon* (Hughes, 1969; Kanal and Chandrasekaran, 1971; Jain and Waller, 1978) Peaking tends to occur later for larger samples. It occurs because too many features overfit the data. Hence, the issue of an optimal feature set is inherently one of classifier design from sample data. Given a sample, among all possible feature sets, which one results in a classifier with minimal error? Or more generally, what is the optimal number of features, in the sense that the expected error for samples of the optimal size is minimized relative to all possible sizes? The answer depends on the type of classifier being designed (Hua et al. 2005).

Since the actual distributions are unknown, evaluating feature sets requires error estimation. Different error estimators will yield different rankings of feature-set performance. Even for large samples, error estimation suffers some imprecision. Thus, if there is a large number of close-to-optimal feature sets arising from a sample, then many of these may be statistically indistinguishable. Moreover, and this seems to be the issue concerning some investigators, different samples will yield different top-performing feature sets. But this is to be expected if each sample yields a list of feature sets (and classifiers) whose error rates are indistinguishable.

Putting aside error estimation for the moment, and focusing solely on classifier design, is it even conceivable to find an optimal feature set for a given sample? In the abstract, one could design a classifier for every possible feature set according to the desired classification rule, find the errors for these, and choose the best classifying feature set. But this program cannot be carried out because the number of possible feature sets to check is astronomical. Moreover, in the absence of prior distributional knowledge, which is the common situation in applications, a full search cannot be avoided if we wish to be assured of finding an optimal feature set (Cover and van Campenhout, 1977). This leads to the feature-selection problem, which is to apply some algorithm to select a less-than-optimal feature set that provides good classification. Depending on the feature-selection algorithm, or possible variant choices within the algorithm, different feature sets will be obtained for the same sample. There is the expectation that the more data one has, the better will be the selected feature set; nonetheless, outcome variability remains. Owing to the instability of feature-selection algorithms, one can surely expect slight changes in the data to result in different outputs. It is important to recognize that feature selection is part of classifier design. It determines the features that serve as variables for the classifier, which is a function on those variables. In doing so, it is part of the classification rule by which the classifier is constructed from the data. If we now incorporate error estimation into our reasoning, not only does it create imprecision in obtaining errors, but it also affects the feature-selection algorithm (Sima et al. 2005).

Given the preceding considerations, we will approach the *optimal-feature-set problem* in the following way: given the class conditional distributions and a positive integer *d*, find the family of all feature sets of size *d* from among the potential features that possess minimal error, and hence are optimal. As posed, feature selection and error estimation do not play a role. Practically, error estimation is a must, so that, regarding the number of “best” feature sets, the issue is not so much one of simply finding the number optimal feature sets, but one of showing that there can be a very large number of feature sets of size *d* whose (true) errors are close to minimal, since these will be statistically indistinguishable. We note in this regard that increasing the sample size does not necessarily mitigate the problem of a large number of statistically indistinguishable feature sets, because as the sample size grows, so too does the size of the feature set producing minimal error, which means extraordinary growth in the number of feature sets of that size.

In the context of gene-expression classification, reflection on biological regulation indicates the enormous gene set variability that can be expected from a microarray study. A set of genes is useful for discriminating pathologies if its behavior, as a collection, varies according to the phenotypes. It may be that the genes involved have some primary regulatory function relating to the phenotype or that they are related, within the overall genome regulation, to other genes governing the phenotype. Given the complex regulatory connectivity within the genome–intricate feedback, massive redundancy, regulatory cascades, and tightly controlled co-regulation of gene cohorts–one should expect a large collection of gene sets having essentially equivalent capability for phenotype discrimination. Add to this the vast number of genes involved. Even if we throw away 80% of the genes on a 20-000-gene microarray, this leaves *C*(4000, *d*) feature sets of size *d,* where *C*(*D*, *d*) denotes the number of subsets of size *d* that can be formed from *D* elements. Even for a modest size *d* this number is enormous. Fore instance, *C*(4000, 40) > 10^80^. Consequently, the errors corresponding to the feature sets can be expected to be extremely dense in the interval [∈, 1], where ∈ is the Bayes error (minimal achievable error) for the classification problem. This observation alone should make it apparent that we can expect to find a large number of feature sets whose errors are statistically indistinguishable from the optimal one.

This paper considers a model in which the features are grouped in such a way that intra-group correlation is substantial whereas inter-group correlation is minimal, the intent being to model the situation in which there are groups of highly correlated co-regulated genes and there is little correlation between the co-regulated groups. This will be accomplished by using a block model for the covariance matrix that reflects these conditions. Focusing on linear discriminant analysis, we demonstrate how these assumptions can lead to large numbers of close-to-optimal feature sets.

## A Covariance Structure Leading to Many Close-to-Optimal Feature Sets

We consider classification for two Gaussian classes. Thus, the classes 0 and 1 possess normal conditional distributions, with mean vectors *μ_k_* and covariance matrices **K***_k_*, for *k* = 0, 1. If we assume that the two classes are equally likely and the covariance matrices are equal, then the optimal classifier is determined by the discriminant *d*_1_(**x**) *– d*_0_(**x**), where

(1) $$d_{k}(\text{x})=-(\text{x}-{\text{u}}_{k}{)}^{\prime }{\text{K}}^{-1}(\text{x}-{\text{u}}_{k})$$

The discriminant is a linear function of **x**, produces hyperplane decision boundaries, and characterizes linear discriminant analysis (LDA). While it is unlikely that the covariance matrices are actually equal, it is this assumption that underlies the optimality of LDA. Moreover, in situations where LDA is applied as a classification rule and sample sizes are limited, such as is often the case with gene-expression data, the assumption of equal covariance matrices is a form of regularization, and is the simplest (and most used) of the various regularized covariance assumptions (Titterington, 1985; Friedman, 1988).

The decision boundary and error for LDA depend on the means, variances, and correlation coefficients. Obtaining direct insight concerning feature selection is not possible in the general case. A standard approach is to adopt a tractable covariance model that represents some assumptions on the relations among the features. For instance, a classic paper on finding the optimal number of features for LDA considers three covariance models (Jain and Waller, 1978). For all models it assumes a common variance *σ*^2^ and a single base value *ρ* from which the correlation coefficients are obtained. The models are defined by their correlation assumptions: (I)*ρ_ij_* = *ρ*,(II)*ρ_ij_* = *ρ*^|*i*–*j*|^ and (III)*ρ_ij_* = *ρ* if |*i−j*| = 1 and *ρ_ij_* = 0 if |*i–j*| > 1. The 3 × 3 forms of these models are given by the following covariance models:

(2) $$\begin{array}{c} \text{I}.\sigma^{2}\;\left[\begin{array}{ccc} 1 & \rho & \rho \\ \rho & 1 & \rho \\ \rho & \rho & 1 \end{array}\right]\;\text{II}\text{.}\;\sigma^{2}\;\left[\begin{array}{ccc} 1 & \rho & \rho^{2}\\ \rho & 1 & \rho \\ \rho^{2}\; & \rho & 1 \end{array}\right]\\ \text{III}\text{.}\;\sigma^{2}\left[\begin{array}{ccc} 1 & \rho & 0\\ \rho & 1 & \rho \\ 0 & \rho & 1 \end{array}\right] \end{array}$$

In this paper we adopt a model suitable to the independent co-regulation of gene groups. Genes in different groups are uncorrelated and those within the same group are correlated. If we label these groups as *G*_1_, *G*_2_,…, *G_m_*, then the covariance matrix is blocked and takes the form

(3) $$\begin{array}{l} \text{K}=\left(\begin{array}{cccc} {\text{B}}_{1} & 0 & \cdots & 0\\ 0 & {\text{B}}_{2} & \ldots & 0\\ \vdots & \vdots & \ddots & \vdots \\ 0 & 0 & \cdots & {\text{B}}_{m} \end{array}\right)\\ {\text{B}}_{k}=\left(\begin{array}{cccc} \sigma_{k1}^{2} & \sigma_{k1}\sigma_{k2}\rho_{k12} & \cdots & \sigma_{k1}\sigma_{kd}\rho_{kld}\\ \sigma_{k2}\sigma_{k1}\rho_{k21} & \sigma_{k2}^{2} & \cdots & \sigma_{k2}\sigma_{kd}\rho_{k2d}\\ \vdots & \vdots & \ddots & \vdots \\ \sigma_{kd}\sigma_{k1}\rho_{kd1} & \sigma_{kd}\sigma_{k2}\rho_{kd2} & \cdots & \sigma_{kd}^{2} \end{array}\right) \end{array}$$

where, for *k* = 1, 2,..., *m*, *σ^2^_ki_* is the variance of the *i*th feature in *G_k_* and *ρ_kij_* is the correlation coefficient for the *i*th and *j*th features in *G_k_*. If **K** has *d* features split into *m* equal-sized blocks, we write **K** ∼ Cov[*d*, *m*]. Special cases of the model result if we assume the blocks take the form of one of the models I, II, or III. What is of interest to us here is that, depending on *ρ* and the variances, it will often be the case that the best feature set of size *m* has one feature from each block, namely, the best feature set is of the form *A* = {*g*_1_, *g*_2_,*…*, *g_m_*}, where *g_k_* ∈ *G_k_* for *k* = 1, 2, ..., *m*. We define a feature set to be *separated* if the features come from different blocks.

**Figure 1.**
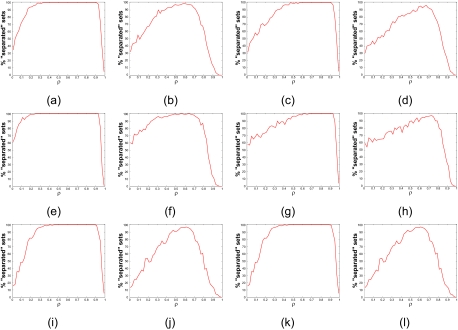
Percentage of separated optimal feature sets as a function of the correlation coefficient *ρ*. First row: **K** ∼ Cov[9, 3], (a) *ρ_ij_* *= ρ*, *σ_ki_* *∼ U*[0.3, 0.47]; (b) *ρ_ij_* *= ρ*, *σ_ki_* ∼ *U*[0.3, 1]; (c) *ρ_ij_* = *ρ*^|^*^i–j^*^|^, *σ_ki_∼U*[0.3, 0.47]; (d) *ρ_ij_* = *ρ*^|^*^i–j^*^|^, *σ_ki_∼ U*[0.3, 1]. Second row: **K** ∼ Cov[10, 2], (e) *ρ_ij_* *= ρ*, *σ_ki_* *∼ U*[0.3, 0.47]; (f) *ρ_ij_* *= ρ, σ_ki_* *∼ U*[0.3, 1]; (g) *ρ_ij_* = *ρ*^|^*^i–j^*^|^, *σ_ki_* *∼ U*[0.3, 0.47]; (h) *ρ_ij_* = *ρ*^|^*^i–j^*^|^, *σ_ki_* *∼ U*[0.3, 1]. Third row: **K** ∼ Cov[10, 5], (i) *ρ_ij_* = *ρ*, *σ_ki_* ∼ *U*[0.3, 0.47]; (j) *ρ_ij_* = *ρ*, *σ_ki_* ∼ *U*[0.3, 1]; (k) *ρ_ij_* = *ρ*^|^*^i–j^*^|^, *σ_ki_* *∼ U*[0.3, 0.47]; (l) *ρ_ij_* = *ρ*^|^*^i–j^*^|^*σ_ki_* *∼ U*[0.3, 1].

We illustrate feature-set separation with several examples. Because the simulations required to construct the examples are extremely computational, we limit ourselves to relatively small covariance matrices. We first consider **K** consisting of three blocks with three features in each, **K** ∼ Cov[9, 3]. Fixing the value of *ρ* throughout **K**, for each simulation the variances are selected from a uniform distribution. We consider two cases: *σ_ki_* ∼ *U*[0.3, 0.47] and *σ_ki_* ∼ *U*[0.3, 1]. The latter case allows a broader range of variances across the features. Fixing *ρ* throughout **K** gives a blockwise model corresponding to model I in (Jain and Waller, 1978) except that it is more realistic in allowing the variances to vary. In all cases 100,000 data points are used to train the best feature set containing 3 features, 50,000 per class, and another 100,000 data points are used for error estimation. The overall process is repeated 10,000 times at different values of *ρ* uniformly distributed between 0 and 1. Figure 1(a) shows the percentage of separated optimal feature sets as a function of *p* for *σ_ki_* *∼ U* [0.3, 0.47]. Except for extreme values of *ρ*, the majority of optimal feature sets are separated, and for 0.3 ≤ *ρ* ≤ 0.9, essentially 100% of the optimal feature sets are separated, indicating that this condition holds if there is at least modest correlation between features within the block and the correlation is not extreme. Note the steep fall off in separation percentage when ρ exceeds 0.9. Figure 1(b), which corresponds *to σ* *_ki_∼ U*[0.3, 1] for the same covariance structure, shows that increased variability results in smaller percentages of separated optimal feature sets; nonetheless, for most of the range of *ρ* there is a significant percentage of separated feature sets. For 0.2 ≤ *ρ* ≤ 0.8, more than 60% of the optimal feature sets are separated.

To demonstrate the commonplace nature of the preceding results, as well as how they can vary depending on the model and the variability of the variance, we have done similar analyses in a number of circumstances. The results are shown in the remaining parts of Fig. 1: (c) **K** ∼ Cov[9, 3], *ρ_ij_* = *ρ*^|^*^i–j^*^|^ (corresponding to model II in (Jain and Waller, 1978)), and *σ_ki_* ∼ *U*[0.3, 0.47]; (d) **K** ∼ Cov[9, 3], *ρ_ij_* *= ρ^|i–j|^* and *σ_ki_* ∼ *U*[0.3, 1]; (e) **K** ∼Cov[10,2], *ρ_ij_* = *ρ*, and *σ_ki_∼U*[0.3, 0.47]; (f) **K** ∼ Cov[10, 2], *ρ_ij_* = *ρ*, and *σ_k_* ∼ *U*[0.3, 1]; (g) **K** ∼ Cov[10,2], *ρ_ij_* = *ρ^|i–j|^* and *σ_ki_* ∼ *U*[0.3, 0.47]; (h) **K** ∼ Cov[10, 2], *ρ_ij_* = *ρ^|i–j|^*, and *σ_ki_* ∼ *U*[0.3, 1]; (i) **K** ∼ Cov[10, 5], *ρ_ij_* = *ρ,* and *σ_ki_* ∼ *U*[0.3, 0.47]; (j) **K** ∼ Cov[10, 5], *ρ_ij_* = *ρ,* and *σ_ki_* ∼ *U*[0.3, 1]; (k) **K** ∼ Cov[10, 5], *ρ_ij_* = *ρ^|i–j|^*, and *σ_ki_* ∼ *U*[0.3, 0.47]; (l) **K** ∼ Cov[10, 5], *ρ_ij_* = *ρ^|i–j|^*, and *σ_ki_* ∼ *U*[0.3, 1]. Even though we have carried out very large simulations, there is still some wobble in the curves.

**Figure 2.**
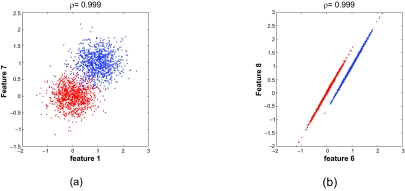
Scatter plots for model **K** ∼ Cov[10, 2],*ρ_ij_* *= ρ,σ_ki_* *∼ U* [0.3, 0.65], with *ρ* = 0.999: (a) feature set {*X1*, *X_7_*}; (b) feature set {X_6_, X_8_}.

To look closer at the low separation percentage when *ρ* ≈ 1, we consider two examples for the model having **K** ∼ Cov[ 10, 2], *ρ_ij_* = *ρ*, *σ_ki_* ∼ *U*[0.3, 0.65]. Let *X*_1_, *X*_2_,…, *X*_10_ denote the features, with *X*_1_, *X*_2_,…, *X*_5_ and *X*_6_, *X*_7_,..., *X*_10_ corresponding to the first and second block of **K**, respectively. In the first example, we let *ρ* = 0.999 and generate one realization of **K** based on the randomly chosen variances. For the resulting **K** the best separated 2-feature set is {*X*_1_, *X*_7_}, with an estimated error of 0.0253. There are nine non-separated feature sets possessing 0 error, {*X*_1_, *X*_3_}, {*X*_1_, *X*_4_}, {*X*_1_, *X*_5_}, {*X*_6_, *X*_8_}, {*X*_6_, *X*_9_}, {*X*_6_, *X*_10_}, {*X*_7_, *X*_8_}, {*X*_7_, *X*_9_}, and {*X*_7_, *X*_10_}, where all error estimates have been obtained using 1,000,000 independent test sample points. Figures 2(a) and 2(b) show 1,000 sample points projected on to features *X*_1_ and *X*_7_, and on to features *X*_6_ and X_8_, respectively. The advantage of {X_6_, X_8_} over {*X*_1_, *X*_7_} is clear.

**Figure 3.**
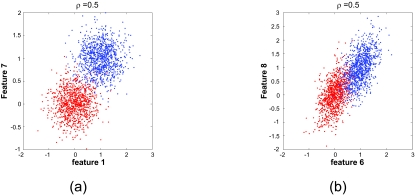
Scatter plots for model **K** ∼ Cov[10, 2], *ρ_ij_* = *ρ, σ_ki_* *∼ U*[0.3, 0.65], with *ρ* = 0.5: (a) feature set {*X*_1_, *X*_7_}; (b) feature set {*X*_6_, *X*_8_}.

In the second example, we let *ρ* = 0.5 and generate one realization of **K** based on the randomly chosen variances. For the resulting **K** the best overall 2-feature set is the separated feature set {X_1_, X_7_}, with an estimated error of 0.0257 (the difference with 0.0253 in the previous case resulting from the variability in error estimation). Figures 3(a) and 3(b) show 1,000 sample points projected on to the features *X*_1_ and *X*_7_, and on to the features *X*_6_ and *X*_8_, respectively. The advantage of {*X*_1_, *X*_7_} over {*X*_6_, *X*_8_}, whose error is 0.0625, is clear.

Comparing Figures 2(b) and 3(b) for the feature set {*X*_6_, X_8_} shows what is happening, *ρ* is the common conditional correlation coefficient for the conditional densities ƒ_X|0_ and ƒ_X|1_. Since *ρ* ≈ 1 in Fig. 2(b), the sample points for each class lie almost on a line, the lines being distinct because the class means are not equal. These two almost linear point masses are easily discriminated by a straight line between them. In Fig. 3(b), where *ρ* = 0.5, we see that the point masses are scattered around the two lines and extensively intermingle. Therefore they are not well discriminated.

## Analysis of Feature-Set Redundancy

Now that we know there are many separated feature sets resulting from the covariance model of Eq. 3, let *A* be such a feature set, *μ*_01_, *μ*_02_,…, *μ*_0_*_m_* and *μ*_11_, *μ*_12_,..., *μ*_1*m*_ denote the means of the features in *A* for classes 0 and 1, respectively, and $\sigma_{1}^{2}\sigma_{2}^{2},\ldots ,\sigma_{m}^{2}$ denote their common variances. By a well-known formula (Duda, et al. 2001), the LDA error for *A* is given by

(4) $$\varepsilon_{A}=\frac{1}{\sqrt{2\pi }}\int_{\text{Δ}(A)/2}^{\infty }e^{-u^{2}/2}du$$

where Δ(*A*) is the Mahalanobis distance. Since *g*_1_ *g*_2_,..., *g_m_* are independent, this distance is determined by

(5) $$\text{Δ}{\left(A\right)}^{2}=\sum_{j=1}^{m}{\left(\frac{\mu_{0j}-\mu_{1j}}{\sigma_{j}}\right)}^{2}$$

Each term of the sum gives the contribution of the feature in reducing the error. The contribution is increased for greater separation of the means and smaller variance. The error tends to 0 as *Δ*(*A*) tends to infinity. If, for any feature *g* with variance $\sigma_{8}^{2}$ and class means *μ*_0*g*_ and *μ*_1*g*_, we let

(6) $$\delta^{2}(g)={\left(\frac{\mu_{0f}-\mu_{1f}}{\sigma_{f}}\right)}^{2}$$

then Eq. 5 can be rewritten as

(7) $$\text{Δ}{\left(A\right)}^{2}=\sum_{j=1}^{m}\delta^{2}(g_{j})$$

To consider feature-set redundancy, suppose *t* is a very small positive number and that, for *k* = 1, 2,..., *m*, the set *G_k_*has a subset $G_{k}^{t}$ = {*g*_*k*1_, *g*_*k*2_,..., *g_km_k__*} such that

(8) $$\delta^{2}(g_{k})-\delta^{2}(g_{ki})<t$$

for *i* = 1, 2,..., *m_k_*. We form a feature set *B* by replacing *q* features in *A* by elements in *q* of the sets $G_{1}^{t},G_{2}^{t},\ldots ,G_{m}^{t}$, *q≤m*. Then, according to Eq. 4, the increase in error resulting from using feature set *B* instead of feature set A is

(9) $$\varepsilon_{\text{B}}-\varepsilon_{\text{A}}=\frac{1}{\sqrt{2\pi }}\int_{\text{Δ}\text{B}/2}^{\text{Δ}(\text{A})/2}{\text{e}}^{-u^{2}/2}du$$

Equations 7 and 8 yield

(10) $$\text{Δ}{\left(A\right)}^{2}-\text{Δ}{\left(B\right)}^{2}<qt$$

Some simple algebra shows that

(11) $$\text{Δ}(B)\;>\;\text{Δ}(A)\;-\;\frac{qt}{\text{Δ}(A)+\sqrt{\text{Δ}{\left(A\right)}^{2}-qt}}$$

Denoting the fraction on the right by *η*(*q, t*), we conclude that

(12) $$\begin{array}{l} \varepsilon_{B}-\varepsilon_{A}=\frac{1}{\sqrt{2\pi }}\int_{\text{Δ}(B)/2}^{\text{Δ}(A)/2}e^{-u^{2}/2}du\\ \;\;\;\;<\frac{1}{\sqrt{2\pi }}\int_{[\text{Δ}(A)-\eta (q,t)]/2}^{\text{Δ}(A)/2}e^{-u^{2}/2}du \end{array}$$

For very small *t*, this difference is negligible, in which case the performances of the two feature sets are negligibly different. Since there are *m_k_* features in *G_k_* satisfying Eq. 8, the *q* elements can be chosen in

(13) $$\nu (q)=\sum_{1\leq k_{1}<k_{2}<\ldots <k_{q}\leq m}m_{k_{1}}m_{k_{2}}\ldots m_{k_{q}}$$

ways. If we consider all feature sets formed by replacing 1,2,..., *m* elements from different blocks, then the total number of close-to-optimal feature sets formed in this manner is

(14) $$\nu =\sum_{q=1}^{m}\nu (q)$$

If the blocks are large and there are several genes in some or all of the blocks satisfying Eq. 8, then this number can be very large.

The point is clear: if the blocks are large, which they may well be when they represent co-regulation of independent cohorts of genes, then there may be many features in a block possessing contributions close to the maximum. These can be interchanged with little effect on the error, thereby leading to close-to-optimal feature sets. In practice, feature sets whose errors are close to optimal will be indistinguishable owing to the need for error estimation.

**Figure 4.**
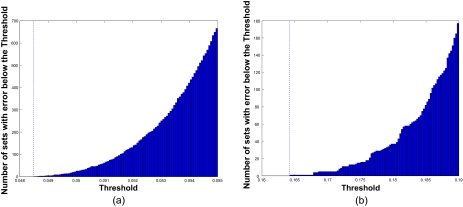
Cumulative histograms of superior feature-set errors: (a) low Bayes error; (b) high Bayes error.

We now illustrate feature-set redundancy with two examples generated from the model of Eq. 3 with **K** ∼ Cov [60, 5] and *ρ_ij_* = *ρ*. In the first example, *ρ* = 0.8 and *σ_ki_* *∼ U*[0.3, 1]. The optimal 4-feature set is separated and has Bayes error 0.0485. Figure 4(a) shows the cumulative histogram of the errors for the superior 4-feature sets. Note that there are almost 700 feature sets whose errors are within 0.007 of the best. This is with 60 possible features. The number would likely be astronomically greater with 40,000 features. In the second example, *ρ* = 0.8 and *σ_ki_* *∼ U*[0.3, 1]. The optimal feature set is separated and has Bayes error 0.1642. Figure 4(b) shows the cumulative histogram of the errors for the superior 4-feature sets. With the larger error, feature-set errors are more spread out, but even in this case there are more than 80 features sets whose errors are within 0.02 of the best.

## Conclusion

Feature-set variability should not be of concern in the context of finding expression-based diagnostic panels. It arises naturally from the relationships among the features, the vast number of feature sets when there is a large number of potential features, and the inherent natures of both classifier design and error estimation from sample data. Owing to the peaking phenomenon, larger sample sizes may not reduce the number of close-to-optimal feature sets because the number of feature sets rises dramatically as their size increases. The important issue is finding good feature sets that can achieve desirable classification rates. Based on what we have seen, it is not unlikely that two studies using comparable technologies and classification procedures will arrive at equally performing feature sets possessing small intersection–or, for that matter, no intersection. Rather than perceive this as a negative, one should see it as a positive. Given the incertitude of feature selection and the imprecision of error estimation, it can be beneficial to have many close-to-optimal feature sets.

Let us close by citing two previous empirical studies that have come to similar conclusions. In one, the authors consider a specific breast-cancer data set and classification with regard to good and bad prognosis (Ein-Dor et al. 2005). Their approach is to rank genes based on their correlation with survival, take feature sets consisting of the first through eighth sets of 70 genes in the list, and to show that classifiers based on each of these feature sets perform essentially the same. In another, it was shown that using an exhaustive search finds large numbers of indistinguishably performing small feature sets (Grate, 2005). By taking a model-based approach we have characterized this phenomenon for linear classification in terms of the discriminant and the actual error, and shown how intra- and inter-group correlation effect the phenomenon.

## References

[b1-cin-02-189] Cover T, van Campenout J (1977). On the possible orderings in the measurement selection problem. IEEE Trans Systems, Man, and Cybernetics.

[b2-cin-02-189] Duda RO, Hart PE, Stork DG (2001). Pattern Classification.

[b3-cin-02-189] Ein-Dor L, Kela I, Getz G, Givol D, Domany E (2005). Outcome signature genes in breast cancer: is there a unique set?. Bioinformatics.

[b4-cin-02-189] Friedman JH (1988). Regularized Discriminant Analysis. Journal of the American Statistical Association.

[b5-cin-02-189] Grate LR (2005). Many accurate small-discriminatory feature subsets exist in microarray transcript data: biomarker discovery. BMC Bioinformatics.

[b6-cin-02-189] Hua J, Xiong Z, Lowey J, Suh E, Dougherty ER (2005). Optimal number of features as a function of sample size for various classification rules. Bioinformatics.

[b7-cin-02-189] Hughes GF (1969). Number of pattern classifier design samples per class. IEEE Trans. Information Theory.

[b8-cin-02-189] Ioannidis JP (2005). Microarrays and molecular research: noise discovery?. Lancet.

[b9-cin-02-189] Jain AK, Waller WG (1978). On the optimal number of features in the classification of multivariate Gaussian data. Pattern Recognition.

[b10-cin-02-189] Jenssen TK, Hovig E (2005). Gene-expression profiling in breast cancer. Lancet.

[b11-cin-02-189] Kanal L, Chandrasekaran B (1971). On dimensionality and sample size in statistical pattern classification. Pattern Recognition.

[b12-cin-02-189] Michiels S, Koscielny S, Hill C (2005). Prediction of cancer outcome with microarrays: a multiple random validation strategy. Lancet.

[b13-cin-02-189] Sima C, Attoor S, Braga-Neto U, Lowey J, Suh E, Dougherty ER (2005). Impact of Error Estimation on Feature-Selection Algorithms. Pattern Recognition.

[b14-cin-02-189] Titterington DM (1985). Common structure of soothing techniques in statistics. International Statistical Review.

